# Marital status and survival in patients with gastric cancer

**DOI:** 10.1002/cam4.758

**Published:** 2016-06-05

**Authors:** Jie‐Jie Jin, Wei Wang, Fa‐Xiang Dai, Zi‐Wen Long, Hong Cai, Xiao‐Wen Liu, Ye Zhou, Hua Huang, Ya‐Nong Wang

**Affiliations:** ^1^ Department of Gastric Cancer and Soft Tissue SarcomaFudan University Shanghai Cancer CenterShanghai200032China; ^2^ Department of OncologyShanghai Medical College, Fudan UniversityShanghai200032China; ^3^ Department of Hepatobiliary SurgeryAffiliated Hospital of Nantong UniversityNantongJiangsu Province226001China

**Keywords:** diagnosis, gastric cancer, marital status, survival, treatment

## Abstract

The objective of this study is to examine the impact of marital status on incidence of metastasis at diagnosis, receipt of surgery, and cause‐specific survival (CSS) in patients with gastric cancer (GC). Research data is extracted from The Surveillance, Epidemiology, and End Results (SEER) database, and 18,196 patients diagnosed with GC from 2004 to 2010 are involved. Effects of marital status on incidence of metastasis at diagnosis, receipt of surgery, and CSS are determined using multivariable logistic regression and multivariable Cox regression models, as appropriate. Single GC patients have a higher incidence of metastasis at diagnosis than married patients, while the differences between divorced/separated patients or widowed patients and married patients are not significant. Among those without distant metastasis, single patients, divorced/separated patients, and widowed patients are much less likely to accept surgery compared with married patients. Finally, in the whole group of 18,196 GC patients, single patients, divorced/separated patients, and widowed patients have shorter CSS compared with married patients, even in each of the TNM stage. Marriage had a protective effect against undertreatment and cause‐specific mortality (CSM) in GC. Spousal support may contribute to higher rate of surgery receipt and better survival in patients with GC.

## Introduction

Globally, the incidence of gastric cancer (GC) ranks the fourth in men and fifth in women among malignancies, and affects more than one million people annually 1. There were 22,220 new cases and 10,990 deaths of GC in the United States estimated by the American Cancer Society in 2014 2. The prognosis for patients with GC is poor with the 5‐year relative survival rates being 29% from 2003 to 2009 in the United States 2. Marriage may have a protective effect on prognosis of cancer patients. Studies indicated that unmarried patients were at higher risk of presentation with metastatic cancer, undertreatment, and shorter survival in various cancer types 3, 4, 5, 6, 7, 8, 9, 10, 11, 12. Mixed 13, 14, 15, 16 or no significant 17, 18, 19 associations between marital status and cancer survival were reported as well. With regard to GC, a recent large population‐based study indicates that individuals who are divorced, widowed, or lived alone are at increased risk for esophagogastric cancer 20. A prospective study showed no evidence of a better 5‐year survival in married patients compared with non‐married patients undergoing surgery for esophageal cancer 19. Until now, little is known about the association between marital status and outcomes of GC.

In this study, we investigated the relation between marital status and incidence of metastasis at diagnosis, receipt of surgery, and CSS in the group of 18,196 GC patients. Data are from the SEER program between 2004 and 2010.

## Methods

### Study population

We extracted clinical data of 18,196 cancer patients with stomach as the single primary site from SEER database. Sponsored by National Cancer Institute, the SEER program collects and publishes incidence, mortality, prevalence, survival, and lifetime risk statistics which can be used to assess the impact of cancer in the general population. The current SEER database consists of 18 population‐based registries, which cover approximately 26% of the United States population 21.

### Patient selection

SEER‐stat software (SEER*Stat 8.1.5) was used for the data extraction and patient selection. The inclusion period was from 2004 to 2010, for the fact that several employed covariates were introduced in the SEER database in 2004 22. Age was limited to 18 years or older, and patients with unknown marital status were excluded. The histologic types consisted of adenocarcinoma, mucinous adenocarcinoma, and signet ring cell carcinoma. The sixth American Joint Classification of Cancer (AJCC) TNM staging system was adopted in this study, and patients with unknown TNM stage were excluded.

### Study variables

According to SEER database, marital status is described as married, single (never married), separated, divorced, and widowed. In this study, the unmarried include single, separated/divorced, and widowed patients. Race/ethnicity is classified as White (non‐Hispanic), Black, Hispanic, and other (American Indian/AK Native, Asian/Pacific Islander, and unknown). The differentiation grades include well/moderately differentiated grade, poorly differentiated/undifferentiated grade, and unknown. Tumor location is classified as cardia and noncardia; noncardia includes fundus, body, greater curve, smaller curve, antrum, and pylorus, according to SEER database. The TNM classification system is defined by the AJCC Cancer Staging Manual (the sixth edition). Types of surgery include gastrectomy with/without regional lymph nodes removed according to the SEER database. Cause‐specific survival is a net survival measure representing survival of a specified cause of death in the absence of other causes of death according to the SEER database. Estimates are calculated by specifying the cause of death. Individuals who die of causes other than the specified cause are considered to be censored. In this study, GC is the specified cause of death.

### Statistical analysis

Baseline patient characteristics were analyzed with chi‐squared test for categorized measurements and Spearman tests for continuous measurements. Multivariable logistic regression was used to determine the association of marital status and incidence of metastasis at diagnosis; the analysis was adjusted for demographic factors (age, sex, and race/ethnicity), tumor location, histological type, differentiated grade, and year of diagnosis. For analysis of receipt of definitive therapy, we excluded patients with metastasis at diagnosis, and 10,013 patients remained eligible. Multivariable logistic regression was used to determine the association between marital status and surgery receipt; the analysis was adjusted for demographic factors (age, sex, and race), tumor location, histological type, differentiated grade, and year of diagnosis. For CSS analysis, multivariable Cox regression analysis was adopted to assess the impact of marital status on CSS after adjustment for demographic factors, TNM stage, histological types, differentiated grades, tumor location, and year of diagnosis. The median follow‐up for the cohort analyzed for CSS was 22 months (range: 1–100). All *P*‐values were two‐sided. The threshold of 0.05 was considered statistically significant. All confidence intervals (CIs) were stated at the 95% confidence level. Statistical analyses were performed using SPSS 19.0.

## Results

### Patient characteristics

Among the cohort of 18,196 patients with GC, 11,114 (61.1%) were married, 2620 (14.4%) were single (never married), 201 (1.1%) were separated, 1523 (8.3%) were divorced, and 2738 (15.1%) were widowed. Eight thousand and one hundred eighty‐three (8183, 44.9%) came up with distant metastasis at diagnosis, and 8580 (41.2%) people accepted surgery for GC. In the whole group, the married were 2.6 years younger than the unmarried which included the single, the separated/divorced, and the widowed (*P* < 0.001). The white and male had a higher percent of being married than other races (black, American Indian/AK Native, Asian/Pacific Islander, and unknown) and females, respectively (*P* < 0.001 for both). The rate of earlier stage (stage I/II) at diagnosis in the married group was lower than the widowed (36.2% vs. 39.7%), but higher than the single (30.3%) and the separated/divorced (35%). Details of patient demographics and pathological features were summarized in Table 1.

**Table 1:** Baseline demographic and tumor characteristics of GC patients in the SEER database

Characteristics	Total	Married	Never married	Divorced/separated	Widowed	*P*
	*N* = 18,196	*N* = 11,114*N* (*%*)	*N* = 2620*N* (*%*)	*N* = 1724*N* (*%*)	*N* = 2738*N* (*%*)	
Sex						<0.001
Male	11,512	7972 (71.7)	1683 (64.2)	990 (57.4)	867 (31.7)	
Female	6684	3142 (28.3)	937 (35.8)	734 (42.6)	1871 (68.3)	
Age^a^						<0.001
18–55 years	4604	2871 (25.8)	1202 (45.9)	470 (27.3)	61 (2.2)	
56–65 years	4246	2879 (25.9)	605 (23.1)	556 (32.3)	206 (7.5)	
66–75 years	4588	3058 (27.5)	434 (16.6)	447 (25.9)	649 (23.7)	
76–85 years	3745	1947 (17.5)	306 (11.7)	210 (12.2)	1282 (46.8)	
86–99 years	1013	359 (3.2)	73 (2.8)	41 (2.4)	540 (19.7)	
Race/ethnicity						<0.001
White(non‐Hispanic)	10,065	6399 (57.6)	1116 (42.6)	974 (56.5)	1576 (57.6)	
Black	2283	950 (8.5)	613 (23.4)	301 (17.5)	419 (15.3)	
Hispanics	3174	1898 (17.1)	607 (23.2)	289 (16.8)	380 (13.9)	
Other^b^	2674	1867 (16.8)	284 (10.8)	160 (9.3)	363 (13.3)	
Year of diagnosis						<0.001
2004–2005	5084	3142 (28.3)	657 (25.1)	472 (27.4)	813 (29.7)	
2006–2007	5222	3207 (28.9)	713 (27.2)	533 (30.9)	769 (28.1)	
2008–2009	5238	3194 (28.7)	809 (30.9)	461 (26.7)	774 (28.3)	
2010	2652	1571 (14.1)	441 (16.8)	258 (15.0)	382 (14.0)	
Tumor location						<0.001
Cardia	6099	4043 (36.4)	799 (30.5)	610 (35.4)	647 (23.6)	
Noncardia	12,097	7071 (63.6)	1821 (69.5)	1114 (64.6)	2091 (76.4)	
Pathologic grade						<0.001
Well/moderate^c^	4330	2616 (23.5)	554 (21.1)	424 (24.6)	736 (26.9)	
Poor/un^d^	11,172	6906 (62.1)	1658 (63.3)	1025 (59.5)	1583 (57.8)	
Unknown	2694	1592 (14.3)	408 (15.6)	275 (16.0)	419 (15.3)	
Histotype						<0.001
Adeno^e^	12,767	7773 (69.9)	1708 (65.2)	1211 (70.2)	2075 (75.8)	
Mucinous^f^	407	240 (2.2)	63 (2.4)	37 (2.1)	67 (2.4)	
Signet^g^	5022	3101 (27.9)	849 (32.4)	476 (27.6)	596 (21.8)	
Metastasis at Dx						<0.001
Yes	8183	4909 (44.2)	1321 (50.4)	814 (47.2)	1139 (41.6)	
No	10,013	6205 (55.8)	1299 (49.6)	910 (52.8)	1599 (58.4)	
TNM stage						<0.001
I	4285	2589 (23.3)	518 (19.8)	393 (22.8)	785 (28.7)	
II	2227	1439 (12.9)	276 (10.5)	211 (12.2)	301 (11.0)	
III	2415	1518 (13.7)	348 (13.3)	207 (12.0)	342 (12.5)	
IV	9269	5568 (50.1)	1478 (56.4)	913 (53.0)	1310 (47.8)	
Surgery						<0.001
Yes	8580	5625 (50.6)	1083 (41.3)	767 (44.5)	1105 (40.4)	
No	9616	5489 (49.4)	1537 (58.7)	957 (55.5)	1633 (59.6)	

Chi‐squared tests were used for categorical measurements. Spearman tests were used for continuous measurements. GC, gastric cancer; SEER, surveillance, epidemiology, and end results; Dx, diagnosis.

a Age was analyzed as continuous measurements (mean: 65 years, median: 66 years, interquartile range: 55 years for Q1, 76 years for Q3), and Spearman tests were used (*r* = 0.205).

b Include American Indian/AK Native, Asian/Pacific Islander, and unknown.

c Highly/moderately differentiated.

d Poorly differentiated/undifferentiated.

e Adenocarcinoma.

f Mucinous cell adenocarcinoma.

g Signet ring cell carcinoma.

### Impact of marital status on incidence of metastasis at diagnosis in GC

Single (never married) GC patients displayed a higher incidence of metastasis at diagnosis than married GC patients (odds ratio [OR] 1.138, 95% CI: 1.040–1.245; *P* = 0.005; Table 2). While difference between the divorced/separated patients and married patients was not significant (*P* = 0.064, Table 2), difference between widowed patients and married patients was not significant either (*P* = 0.085, Table 2). Black GC patients had a lower incidence of metastasis at diagnosis compared with the white (OR 0.825, 95% CI: 0.748–0.911; *P* < 0.001; Table 2). Year of diagnosis had no significant impact on incidence of metastasis at diagnosis. Patients with cardia cancer had a lower rate of distant metastasis compared with noncardia GC patients (OR 0.716, 95% CI: 0.667–0.769; *P* < 0.001; Table 2). Poorly differentiated/undifferentiated GC patients had a higher incidence of metastasis compared with the well/moderately differentiated GC (OR 1.428, 95% CI: 1.322–1.543; *P* < 0.001; Table 2). GC patients with signet ring cell carcinoma and mucinous cell adenocarcinoma had a lower incidence with metastasis compared with patients with adenocarcinoma (OR 0.621, 95% CI: 0.502–0.768, *P* < 0.001; OR 0.775, 95% CI: 0.720–0.835, *P* < 0.001; respectively).

**Table 2:** Impact of marital status on diagnosis and treatment of gastric cancer

Variables	Metastatic disease at Dx		Surgery performed or not^2^	
	OR^1^ (95% CI)	*P*	OR^2^ (95% CI)	*P*
Sex	NI		NI	
Male vs. Female				
Age^1^	0.983 (0.981–0.985)	<0.001	0.962 (0.958–0.966)	<0.001
Race/ethnicity				
White (non‐Hispanic)	Reference		Reference	
Black	0.825 (0.748–0.911)	<0.001	0.769 (0.657–0.899)	0.001
Hispanic	1.040 (0.954–1.134)	0.368	0.903 (0.781–1.044)	0.167
Other^a^	0.681 (0.620–0.747)	<0.001	1.469 (1.257–1.716)	<0.001
Year of diagnosis				
2004–2005	Reference		Reference	
2006–2007	1.045 (0.964–1.131)	0.286	1.026 (0.900–1.169)	0.700
2008–2009	0.973 (0.898–1.054)	0.554	0.850 (0.749–0.965)	0.012
2010	1.089 (0.988–1.200)	0.080	0.747 (0.641–0.871)	<0.001
Tumor location				
Noncardia	Reference		Reference	
Cardia	0.716 (0.667–0.769)	<0.001	0.441 (0.395–0.493)	<0.001
Differentiated grade				
Well/moderate^b^	Reference		Reference	
Poor/un^c^	1.428 (1.322–1.543)	<0.001	1.044 (0.932–1.170)	0.457
Unknown	3.403 (3.066–3.777)	<0.001	0.197 (0.166–0.234)	<0.001
Histological type				
Adenocarcinoma	Reference		Reference	
Mucinous cell adenocarcinoma	0.621 (0.502–0.768)	<0.001	2.627 (1.813–3.806)	<0.001
ignet ring cell carcinoma	0.775 (0.720–0.835)	<0.001	0.996 (0.880–1.127)	0.996
Marital status				
Married	Reference		Reference	
Single (never married)	1.138 (1.040–1.245)	0.005	0.559 (0.482–0.647)	<0.001
Divorced/separated	1.080 (0.972–1.199)	0.064	0.681 (0.576–0.806)	<0.001
Widowed	1.086 (0.989–1.192)	0.085	0.571 (0.499–0.653)	<0.001

OR^1^ adjusted for demographics (age and race), tumor location, differentiated grade, histological type, and year of diagnosis. OR^2^ adjusted for demographics (age and race), tumor location, differentiated grade, histological type, and year of diagnosis. NI: not included in the bivariate logistic regression analysis. HR, hazard ratio; OR, odds ratio; CI, confidence interval.

^1^Age was analyzed as continuous measurements in both analyses.

^2^Exclude patients with metastatic disease. The event for modeling surgery performed or not is “surgery performed”.

a Include American Indian/AK Native, Asian/Pacific Islander, and unknown.

b Highly / moderately differentiated.

c Poorly differentiated / undifferentiated.

### Impact of marital status on receipt of surgery in GC

To determine the differences in receipt of surgery according to marital status, we excluded patients with metastasis at diagnosis. In the rest of 10,013 cases, the unmarried were less likely to accept surgery, the ORs and 95% CIs are as follows: 0.559 (0.482–0.647) for the single, 0.681 (0.576–0.806) for the separated/divorced, 0.571 (0.499–0.653) for the widowed (Table 2). Adjustment was performed with patients’ demographics (age and race/ethnicity), tumor location, differentiated grade, histological type, and year of diagnosis. In addition, we found that black GC patients had a lower probability to accept surgery than the white, and patients diagnosed between 2008 and 2009 or in 2010 were more likely to accept surgery than those diagnosed between 2004 and 2005 (OR 0.850, 95% CI: 0.749–0.965, *P* = 0.012; OR 0.747, 95% CI: 0.641–0.871, *P* < 0.001; respectively, Table 2). Patients with cardia cancer were much less likely to accept surgery compared with noncardia GC patients (OR 0.441, 95% CI: 0.395–0.493, *P* < 0.001, Table 2).

### Impact of marital status on CSS in GC

**Figure 1:**
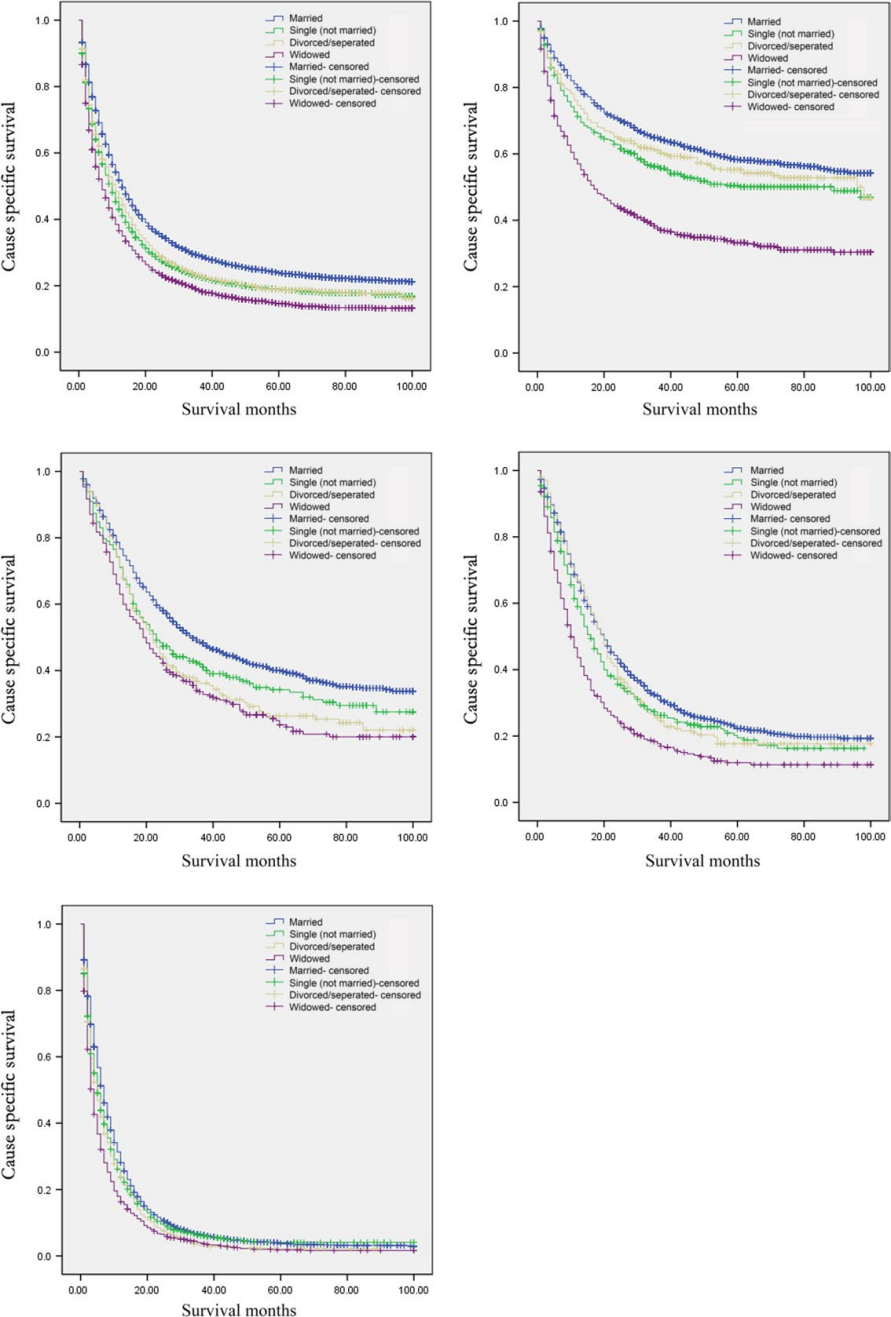
Survival curves in 18,196 patients according to marital status. (A) Stage I‐IV, χ2 = 291.817, *P* < 0.001. (B) Stage I, χ2 = 224.840, *P* < 0.001. (C) Stage II, χ2 = 44.194, *P* < 0.001. (D) Stage III, χ2 = 62.773, *P* < 0.001. (E) Stage IV, χ2 = 149.243, *P* < 0.001.

With regard to the association between marital status and CSS of GC patients, Cox proportional hazards regression model was adopted in total of 18,196 GC cases. Analysis was adjusted with patients’ demographics (sex, age and race/ethnicity), tumor location, differentiated grade, histological type, tumor stage, and year of diagnosis. Results showed that married patients enjoyed longer CSS time than the unmarried, and differences were significant with the hazard ratios and 95% CIs as follows: 1.279 (1.216–1.344) for the single (never married), 1.217 (1.149–1.290) for the separated/divorced, 1.274 (1.209–1.342) for the widowed (Table 3). Further analysis was conducted according to TNM stage, the association between marital status and CSS length remained significant as well. Details were shown in Table 4 and Figure 1. Among other clinical parameters, we found that female GC patients had better CSS than the male GC patients (OR 0.956, 95% CI: 0.920–0.992, *P* = 0.018, Table 4). GC patients’ diagnosis between 2008 and 2009 or in 2010 had better CSS than those diagnosis between 2004 and 2005 (OR 0.871, 95% CI: 0.833–0.910, *P* < 0.001; OR 0.876, 95% CI: 0.828–0.926, *P* < 0.001; respectively, Table 4). Poorly differentiated/undifferentiated GC patients displayed worse CSS than well/moderately differentiated GC patients (OR 1.225, 95% CI: 1.172–1.281, *P* < 0.001, Table 4). GC patients diagnosed with mucinous cell adenocarcinoma displayed better CSS than those diagnosed with adenocarcinoma (OR 0.825, 95% CI: 0.734–0.928, *P* = 0.001, Table 4). GC patients at stage II/III/IV had significantly worse CSS compared with those at stage I (details at Table 4).

**Table 3:** Impact of marital status on CSS of gastric cancer

Variables	CSS	
	HR^3^ (95% CI)	*P*
Sex		
Male	Reference	
Female	0.956 (0.920–0.992)	0.018
Age*	1.018 (1.017–1.020)	<0.001
Race		
White	Reference	
Black	1.049 (0.994–1.107)	0.079
Hispanic	0.969 (0.924–1.017)	0.205
Other	0.773 (0.733–0.816)	<0.001
Year of diagnosis		
2004–2005	Reference	
2006–2007	0.913 (0.874–0.953)	<0.001
2008–2009	0.871 (0.833–0.910)	<0.001
2010	0.876 (0.828–0.926)	<0.001
Tumor location		
Noncardia	Reference	
Cardia	1.007 (0.968–1.048)	0.728
Differentiated grade		
Well/moderate^b^	Reference	
Poor/un^c^	1.225 (1.172–1.281)	<0.001
Unknown	1.420 (1.341–1.503)	<0.001
Histological type		
Adenocarcinoma	Reference	
Mucinous cell adenocarcinoma	0.825 (0.734–0.928)	0.001
Signet ring cell carcinoma	1.053 (1.012–1.097)	0.012
Stage		
I	Reference	
II	1.513 (1.412–1.621)	<0.001
III	2.272 (2.130–2.424)	<0.001
IV	5.445 (5.171–5.734)	<0.001
Marital status		
Married	Reference	
Single (never married)	1.279 (1.216–1.344)	<0.001
Divorced/separated	1.217 (1.149–1.290)	<0.001
Widowed	1.274 (1.209–1.342)	<0.001

HR^3^ adjusted for demographics (age, sex, and race), tumor location, histological type, differentiated grade, stage, and year of diagnosis. CSS, cause‐specific survival; CI, confidence interval; HR, hazard ratio.

*Age was analyzed as continuous measurement.

^b^Highly / moderately differentiated.

^c^Poorly differentiated / undifferentiated.

**Table 4:** Marital status on cause‐specific survival (CSS) of gastric cancer based on different cancer stages in 18,196 patients

TNM stage	5‐year CSS	Multivariate analysis	
		HR^4^ (95% CI)	*P*
Stage I			
Married	58%	Reference	
Single (never married)	50%	1.448 (1.255–1.670)	<0.001
Divorced/separated	55%	1.242 (1.053–1.465)	0.010
Widowed	33%	1.453 (1.284–1.645)	<0.001
Stage II			
Married	40%	Reference	
Single (never married)	34%	1.322 (1.121–1.559)	0.001
Divorced/separated	26%	1.340 (1.121–1.602)	0.001
Widowed	24%	1.209 (1.023–1.430)	0.026
Stage III			
Married	22%	Reference	
Single (never married)	19%	1.302 (1.134–1.496)	<0.001
Divorced/separated	18%	1.134 (0.960–1.339)	0.140
Widowed	12%	1.240 (1.071–1.434)	0.004
Stage IV			
Married	4%	Reference	
Single (never married)	4%	1.210 (1.137–1.287)	<0.001
Divorced/separated	2%	1.208 (1.124–1.298)	<0.001
Widowed	2%	1.211 (1.131–1.298)	<0.001

HR^4^ adjusted for demographics (sex, age and race), tumor location, histological type, and differentiated grade, and year of diagnosis. CI, confidence interval; HR, hazard ratio.

## Discussion

In this study, we find that marriage has a protective effect on GC patients. Married GC patients have a lower incidence of metastasis at diagnosis than single patients. Married GC patients are more likely to accept surgery than the single, the divorced/separated, and the widowed. In addition, married patients have a lower GCSM than the unmarried, even in each of the TNM stage. It is the first study to demonstrate the significant protective impact that marriage can have on incidence of metastasis at diagnosis, surgery receipt, and CSS of GC patients.

Incidences of metastasis at diagnosis in each group are as follows: 44.2% for the married, 50.4% for the single (never married), 47.2% for the separated/divorced, 41.6% for the widowed. The incidences of metastasis in each group may be affected by age, a previous study demonstrated that GC tends to exhibit more aggressive tumor behavior in young patients (40 years or younger) than in old patients 23. After adjusted for age, race, tumor location, differentiated grade, histological type, and year of diagnosis, only single patients displayed a higher incidence of metastasis than married GC patients; differences between the separated/divorced or the widowed and the married are not significant. To explain this phenomenon, married people may have better access to care than the unmarried 24. Reports have demonstrated that even in nations with universal access to free care, sociodemographic factors influence outcomes in various health conditions 24, 25, 26, 27. Additionally, married people may benefit from encouragement by spouses to seek medical attention for worrisome symptoms.

The association between marital status and the receipt of surgery is valid in our study in GC cases without distant metastasis. Spouses of these married patients may encourage them to perform surgery versus expectant management 28, which could partly account for the discrepancies. Studies showed diagnosis of cancer caused more distress than other diseases 29. Married people were easier to benefit from social support from their friends and family and displayed less distress and depression after the cancer diagnosis 30. Patients with depression displayed three times greater odds to be noncompliant with medical treatment recommendations compared with those who were not depressed 31. And a study in breast cancer demonstrated that women patients with depression were less likely to accept surgery 32. Physicians should pay more attention to those unmarried and diagnosed with GC, and recommen them for psychologist's help if necessary. Adequate support and timely psychological interference may contribute to more possibility of receiving surgery in unmarried GC patients.

Partly resulting from the advantage in treatment selection, the married enjoy a much better CSS than those unmarried. There are explanations for the survival advantage in other aspects. Studies suggested that the unmarried may be at greater risk of smoking and alcohol use 33, 34, which could do additional harm to the patients’ health. Physiologically, abnormal diurnal cortisol rhythm predicts earlier cancer death 35, 36, 37, and the abnormal profiles might be associated with quality of social support from friends and marriage. Suppression of natural killer (NK) cell count and NK function may be involved in the progression 38. Adverse results exist 39 and further investigations on this subject are warranted.

This study gives conclusive results of the association between marital status and outcomes of GC. There are some potential limitations we should consider. Firstly, risk factors included in this study is limited. Risk factors such as smoking, alcohol consumption, above normal body weight, high salt/fat consumption, low vegetable and fruits consumption, low economic status, other chronic gastric diseases, and HP infection are not recorded in the SEER database 40, 41. Yet, health behavior variables including smoking, diet, and physical activity, were reported to have no indirect effect on the association between living arrangements and mortality 42. Secondly, some unmarried patients may cohabit with a partner other than a spouse which could provide support to the patients. Data from the 2010 US Census indicate that about 90 million unmarried Americans more than 15 years old live “with other persons”, whereas, only approximately 30 million live alone 3. Neglect of the cohabiting patients may lessen the variation in mortality. Thirdly, information on comorbidities besides GC is not available from the SEER database’ this is a possible limitation to this study.

Despite the stated limitations, our study demonstrates that, unmarried GC patients are less likely to accept surgery and have worse CSS than married GC patients. Spousal support may contribute to higher rate of surgery receipt and better survival in GC. Special attention should be paid to the unmarried GC patients; social support may help improve their prognosis.

## Conflict of Interest

None.
